# Analyzing Engagement: A Study of Teething-Related Discussions and Trends on Facebook

**DOI:** 10.7759/cureus.69107

**Published:** 2024-09-10

**Authors:** Hadeel Alhumaidan, Sanaa N Al-Haj Ali

**Affiliations:** 1 Department of Orthodontics and Pediatric Dentistry, College of Dentistry, Qassim University, Buraydah, SAU

**Keywords:** health care, health risk, infant, social media, teething‎

## Abstract

Background: Caregivers often use Facebook to seek advice on children's health issues, including teething. However, the lack of professional oversight on this platform can lead to the spread of misinformation, emphasizing the need for research on user engagement with teething content.

Aim: This longitudinal study assesses public interaction with teething-related information on Facebook, aiming to inform children's health organizations about creating accurate social media content.

Methods: A year-long analysis of English-language Facebook posts tagged "teething" was conducted, focusing on public pages and groups, while excluding unrelated or private content. Sources were categorized, and engagement was measured against page likes or group memberships. Chi-square and Kruskal-Wallis tests were used to evaluate statistical differences in post types and engagement by source (P < 0.05).

Results: Out of 193 relevant posts, interactive content was the most common (112 posts (58%)), followed by miscellaneous (59 posts (30.6%)) and educational content (22 posts (11.4%)). Noticeable confusion among users was observed concerning teething symptoms and processes. Misinformation was prevalent, particularly regarding the use of amber necklaces, amber teething toys, topical anesthetics, and nocturnal bottle feeding for relief. The engagement rate for teething content was low at 1.09%, with significant variations in content strategies by source (P < 0.001), though engagement rates were consistent (P = 0.406).

Conclusions: The study uncovers active but misinformed discussions about teething on Facebook, marked by a lack of evidence-based advice and low engagement rates. These findings emphasize the immediate need for children's health organizations to provide scientifically accurate information to foster a better-informed online community.

## Introduction

In the digital age, social media platforms have revolutionized the dissemination of health-related information, with Facebook's vast network playing a crucial role in health communication [1]. This platform, due to its unparalleled user base, has become a particularly influential medium, allowing organizations and individuals to connect with dispersed audiences like never before [2-5]. Its myriad of interactive tools, including the "Groups" feature, provides a dedicated space for users to engage in discussions about shared interests, which significantly includes health-related topics [4,6].

Teething, or the eruption of primary teeth, is a natural phase that begins around six months of age and continues until about three years old [7,8]. This period can provoke a range of symptoms, from mild discomfort to more pronounced irritability and low-grade fever, leading caregivers to seek support and information through online communities [2,9].

The American Academy of Pediatric Dentistry endorses guidelines for symptom management, which recommend safe practices such as oral analgesics and teething rings, while advising against potentially harmful treatments such as topical anesthetics or homeopathic remedies [10]. However, teething-related advice found on Facebook groups and pages can vary greatly in its accuracy, and reliance on such information can propagate misinformation, potentially leading to harmful health practices for infants [8]. Of particular note is the increased attention on social media platforms given to the use of amber necklaces for teething children, despite being steeped in myths about amber’s alleged pain-reducing benefits [11].

Amber stone spheres, often fashioned into necklaces or bracelets, are believed to release succinic acid upon contact and heat, which when absorbed by the skin, is thought to create local analgesic and anti-inflammatory effects. Manufacturers of these products advocate for their continuous and prolonged use to achieve the desired therapeutic effect [12]. However, the use of such products brings imminent health risks for children, including choking, strangulation, swallowing of the stone beads, and bacterial infections in the skin and/or oral cavity, in addition to a lack of evidence supporting their benefits [12].

The situation further deteriorates when parental misconceptions about teething symptoms result in mismanagement of childhood illnesses, with severe conditions occasionally misattributed to teething [13]. For instance, symptoms such as fever, irritability, and loss of appetite, if wrongly attributed to teething, could leave significant conditions such as primary herpetic infection (primary herpetic gingivostomatitis) undiagnosed and untreated [14]. It underscores an urgent need for research into the exchange of misinformation on social media, the nature of user engagement with such content, and the consequential health outcomes [1].

Therefore, a thorough examination of user engagement with teething content on Facebook is paramount. Engagement metrics such as likes, shares, and comments, being visible to anyone with access to the posted content, serve as indicators of content's popularity and reach, reflecting its potential impact on public health perceptions and caregiver behaviors [1,6]. This study aims to critically assess public engagement with teething information on Facebook, pinpoint drivers of high engagement, and support healthcare professionals in creating evidence-based social media content. The insights gained will contribute to public health strategies and policy, promoting the dissemination of accurate health information and improving pediatric oral health outcomes.

## Materials and methods

In this longitudinal study, a dedicated Facebook account was established in April 2022 to identify and analyze content related to teething. To locate relevant content, a keyword search was employed using the sole term "teething." This search strategy was based on a previously validated method [3]. The account was configured with minimal personal information (only a name and date of birth), devoid of any Facebook "Friends", and lacked any prior Facebook "Likes". To mitigate the risk of search results being skewed by prior activity or network bias, all cookies were cleared, and location services were disabled before conducting searches.

Over the course of one year, from May 2022 to May 2023, posts on Facebook pages and groups pertinent to teething were systematically documented. Quantitative engagement metrics for each post, including likes, comments, and shares, were recorded. Inclusion criteria excluded pages and groups not directly related to teething, those with restricted access, content not in English, inactive entities, and any posts falling outside the defined study period. Demographic information regarding the origin country, the date of page or group creation, and the source of the post was also gathered.

The sources of the posts were classified into four distinct categories: professional organizations (defined as societies, associations, or organizations affiliated with any field in medicine or dentistry), dental or health companies (commercial entities associated with dental or health products or services), academic institutions (universities or dental/medical schools), and individual users (Facebook users without affiliations to the aforementioned entities).

Additionally, the number of likes for each page and the number of members for each group were noted. Two researchers independently analyzed the eligible posts, categorizing them into one of five subject areas, consistent with previous studies: [3,15] academic (articles or links to articles published in an academic journal), news (information published in a nonacademic source), educational (used mainly as a teaching tool for the general public), interactive (requesting user input, including possible diagnosis of a clinical case), or miscellaneous (event updates and advertising, personal highlights, and holiday greetings).

The engagement rate for each post was computed by summing the number of likes, comments, and shares, which was then normalized by the number of likes on the page or the number of group members, as appropriate [3,15].

Statistical analysis was conducted with Statistical Product and Service Solutions (SPSS; IBM SPSS Statistics for Windows, Armonk, NY) software, from which descriptive statistics were derived. The association between the types of posts and the specific Facebook pages or groups was assessed using the chi-square test. Furthermore, the Kruskal-Wallis test was employed to evaluate the significance of differences in engagement rates among various Facebook pages and groups. A P value below 0.05 was considered indicative of statistical significance.

## Results

**Figure 1:**
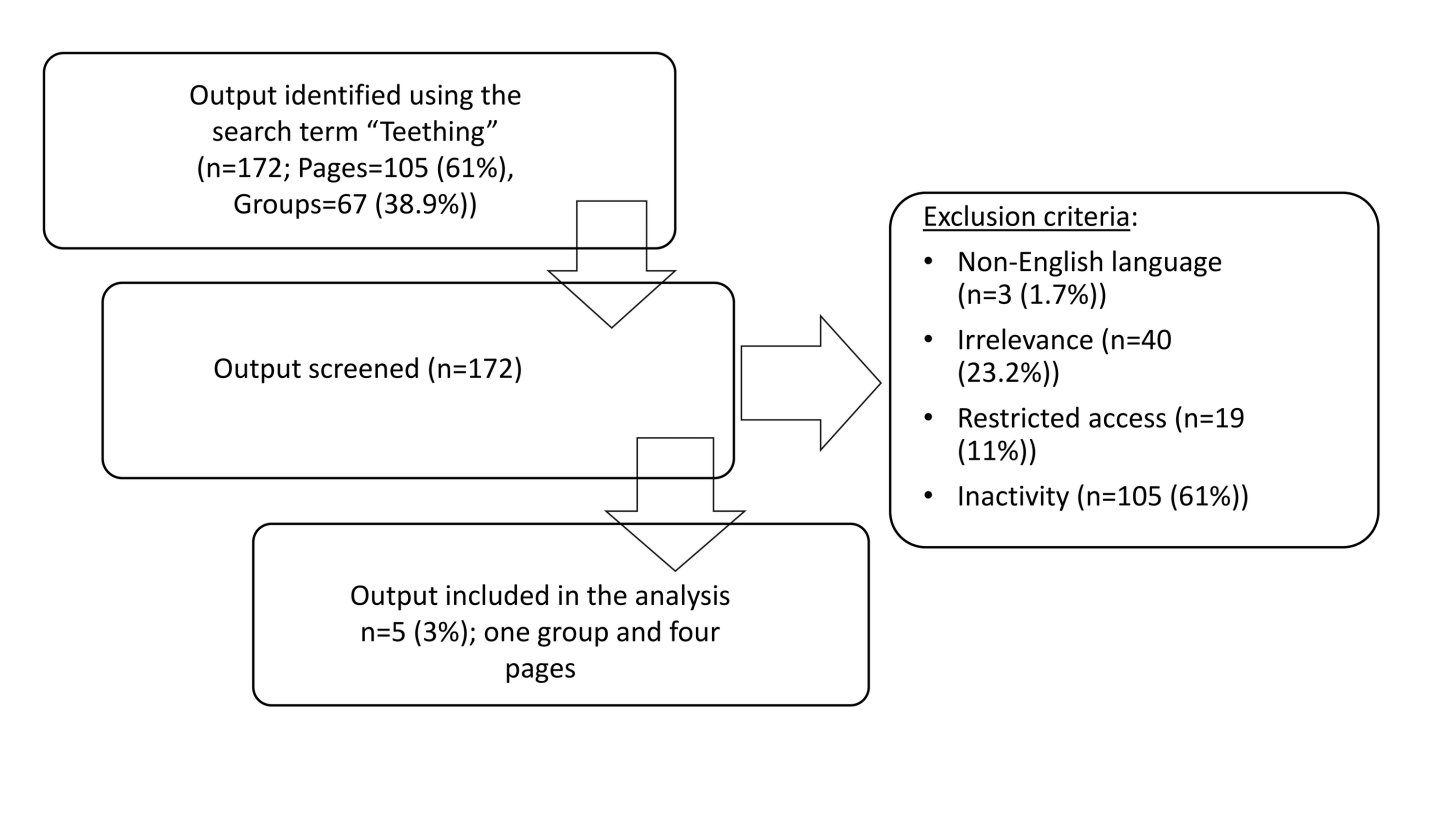
Facebook search strategy and results

The term "teething" was searched on Facebook, resulting in a total of 172 groups and pages, of which 67 were groups (38.9%) and 105 were pages (61%). After applying the inclusion criteria, only one group and four pages were found to be suitable for the study (as detailed in Table 1), leading to the exclusion of the remaining groups and pages (as illustrated in Figure 1).

**Table 1:** Overview of Facebook pages and groups analyzed in the study

Page/group name	Country of origin	Source of upload	Date created	Likes/members (N)
Baby teething*	USA	Individual user	March, 2017	4000
The teething egg **	USA	Dental/health company	October, 2015	118000
Prodol**	South Africa	Dental/health company	December, 2019	9700
The teething company **	USA	Dental/health company	February, 2022	79
Gummee teething**	Malta	Individual user	April, 2020	525
*Facebook group, **Facebook page				

A total of 193 posts were analyzed and categorized, with the majority being interactive (112 posts, representing 58% of the total). Miscellaneous posts accounted for 30.6% (59 posts), and educational posts made up 11.4% (22 posts). Notably, no news or academic posts were identified in the analyzed data.

**Figure 2:**
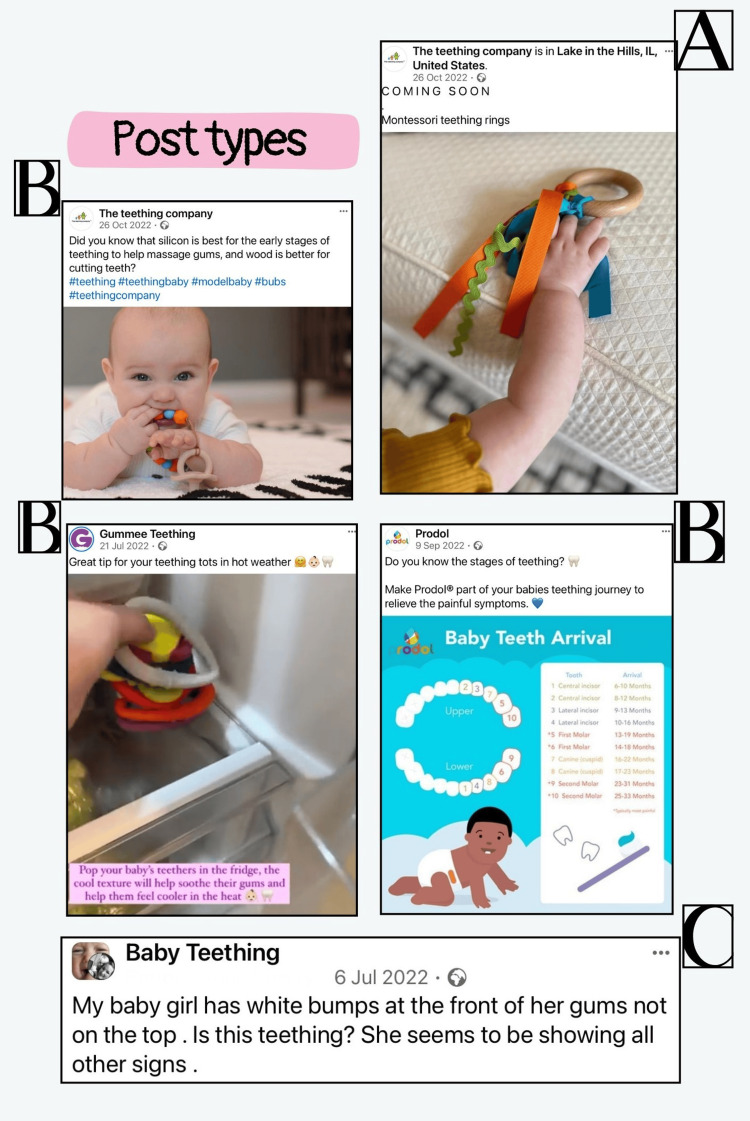
Examples of different post types (A: miscellaneous post, B: educational post, and C: interactive ‎post)‎

Figure 2 showcases examples of different post types. The posts were further classified based on their primary focus. The interactive posts largely concentrated on three areas: teething relief and products (61 posts, 54.5% of interactive posts), teething signs and symptoms (31 posts, 27.6%), and the timing and sequence of teething (20 posts, 17.9%). Users concerns about the timing and sequence of teething included inquiries about the typical age range for the onset of teething, expressing concerns when teeth had not appeared between two months and one year of age, and seeking advice on how to facilitate the beginning of teething (reflected in 12 posts (10.7% of interactive posts)). They also inquired about the expected duration from when symptoms first appear to when a primary tooth erupts (reflected in four posts (3.5% of interactive posts)), and the standard order of tooth eruption, including the sequence of anterior versus posterior teeth, and whether the upper or lower primary teeth typically appear first (reflected in four posts (3.5% of interactive posts)).

The discussions on teething signs and symptoms spanned a variety of issues, including loss of appetite, vomiting, fever, excessive drooling, sleep disturbances, constant chewing or crying, and unexplained weight loss, as mentioned in 25 posts (22.3% of interactive posts). Additionally, two posts (1.7% of interactive posts) indicated user confusion in distinguishing between teething symptoms and those of a common cold. In four posts (3.5% of interactive posts), users shared photos of their children's gums, seeking community assistance to identify if white bumps or oral/perioral rashes were indicative of teething.

Teething relief and products were prominent themes in the interactive posts analyzed. Users actively shared experiences and sought advice on various methods to alleviate teething discomfort. The content featured oral analgesics in nine posts (8% of interactive posts) and topical anesthetics in three posts (2.6% of interactive posts). Additionally, amber necklaces were discussed in two posts (1.7% of interactive posts), and nocturnal bottle feeding was suggested in another (0.9% of interactive posts). The majority of the posts included positive reviews of teething products, with 46 posts (41% of interactive posts) showcasing photos or videos of children using these products, often accompanied by requests for opinions or feedback.

The miscellaneous post category was dominated by advertisements for teething products (56 posts, 94.9% of miscellaneous posts). Among these, the "Prodol" page, which focuses on a commercial topical anesthetic, contributed to six posts (10.1% of miscellaneous posts). "The Teething Egg" page was also notable, with 10 posts (17% of miscellaneous posts) dedicated to its teething toys, which are characterized by the inclusion of amber. Personal highlights appeared in three posts of the miscellaneous category (5%).

The educational post category included 22 posts (11.4% of posts), most of which educated users about the indications for teething products, described their features, or explained how to apply them. Among these, one post (4.5% of educational posts) specifically detailed the chronology of primary tooth eruption.

In terms of Facebook page/group activity, the Facebook page “Gummee Teething” was the most active with the majority of the posts (99 posts, 51.3% of total posts), followed by the Facebook group "Baby Teething" with 62 posts (32.1% of total posts) and the Facebook page "Prodol" with 13 posts (6.7% of total posts). Tables 2-3 summarize the monthly post counts and engagement rates for the included Facebook page and groups. "Gummee Teething" had the highest average engagement rate at 2.68%, followed by "The Teething Company" at 2.6%. The overall engagement rate was 1.09%.

**Table 2:** Number of posts and engagement rate per month for Facebook pages and groups included in the ‎study

	May 2022	June 2022	July 2022	August 2022	September 2022	October 2022
Baby teething*	7 (0.14)	21 (0.2)	5 (0.29)	8 (0.18)	7 (0.12)	7 (0.1)
The teething egg **	3 (0.006)	2 (0.007)	0 (0)	0 (0)	0 (0)	0 (0)
Prodol**	4 (0.042)	2 (0.054)	1 (0.037)	1 (0.02)	2 (0.005)	1 (0.02)
The teething company **	3 (10.5)	1 (2.53)	0 (0)	2 (8.86)	0 (0)	3 (9.28)
Gummee teething**	27 (0.81)	4 (0.95)	5 (2.59)	12 (1.17)	4 (5.52)	2 (1.23)
*Facebook group, **Facebook page						

**Table 3:** Number of posts and engagement rate per month and in total for Facebook pages and groups ‎included in the study

	November 2022	December 2022	January 2023	February 2023	March 2023	April 2023	Total
Baby teething*	2 (0.15)	1 (0.12)	0 (0)	1 (0.075)	2 (0.125)	1 (0.2)	62 (0.142)
The teething egg **	1 (0.003)	4 (0.003)	0 (0)	0 (0)	0 (0)	0 (0)	10 (0.0015)
Prodol**	0 (0)	0 (0)	2 (0.004)	0 (0)	0 (0)	0 (0)	13 (0.0152)
The teething company **	0 (0)	0 (0)	0 (0)	0 (0)	0 (0)	0 (0)	9 (2.60)
Gummee teething**	4 (0.38)	4 (0.24)	7 (14.58)	5 (0.76)	13 (1.15)	12 (4.65)	99 (2.69)
*Facebook group, **Facebook page							

In terms of statistical analysis, the Kruskal-Wallis test revealed no statistically significant differences in engagement rates across the pages and groups (P = 0.406). Conversely, the chi-square test showed a statistically significant association between post types and specific Facebook pages/groups (P < 0.001). "Baby Teething" focused predominantly on interactive posts (61 posts (98.4%)), "Prodol" and Gummee Teething” had a more balanced distribution, and "The Teething Company" and “The Teething Egg” primarily featured miscellaneous posts (seven to ten posts each (77.8-100%)).

## Discussion

The empirical investigation into Facebook's role in distributing teething-related information ‎highlights the multifaceted nature of health communication within the digital space [6]. A notable shortfall in content that originates from professional healthcare sources was found, with ‎searches yielding content that did not align with professional healthcare advice.

In dissecting 193 posts, it was observed that more than half of the posts were interactive. This ‎underscores the role of Facebook groups as hubs for community support [2], where users ‎actively discuss signs, symptoms, eruption timing and sequence, as well as relief strategies for ‎teething. This level of engagement reflects a significant demand for information and assistance ‎in managing the teething process, which is often misunderstood and confused with symptoms of ‎unrelated illnesses such as oral rashes and weight loss [7,11].

The miscellaneous content category, comprising 30.6% of the posts, was primarily ‎commercial, promoting products such as "Prodol," a topical anesthetic with benzocaine not ‎recommended for teething, along with various teething toys. Some posts controversially ‎claimed that amber could relieve teething pain, despite its unproven benefits and potential ‎safety risks [8,12]. Furthermore, certain interactive posts endorsed inappropriate remedies such as ‎overnight bottle feeding to soothe nighttime crying, a practice associated with early childhood ‎caries [16]. The high volume of posts about teething products and remedies underscores the ‎crucial need for educational content regarding safe and effective teething practices.

Educational posts accounted for only 11.4% of the total content, significantly less than ‎interactive and miscellaneous posts, and their primary focus was on the indications, features, ‎and application methods of teething toys. A significant concern is the absence of educational ‎content on teething authored by health professionals or recognized children's organizations such as ‎the American Academy of Pediatrics or the American Academy of Pediatric Dentistry. This ‎absence raises concern about the accuracy of the information presented. While these posts ‎engage users, their educational value is compromised by a lack of professional oversight and a ‎narrow focus on product features, which does not adequately address user queries about teething ‎or correct any misinformation. This underscores the urgent need to scrutinize the sources of ‎health-related information on Facebook to ensure they adhere to established standards of ‎accuracy and reliability. It is crucial for healthcare professionals and children's organizations to ‎actively provide credible, educational content about teething on Facebook. Furthermore, ‎platforms must enhance content moderation efforts to prevent the spread of misinformation ‎‎[6,11].

The user engagement analysis revealed a modest average engagement rate of 1.09%, hinting ‎at passive interaction with teething-related content. This could be attributed to the lack of ‎specialized teething groups and a dearth of educational material from a reputable source. It is also ‎because of narrower audience demographics, as mothers or primary caregivers of infants are the ‎primary target group. Comparable ‎trends of low engagement have been noted for other health ‎topics on Facebook, such as ‎diabetes mellitus [4], dental trauma [15], and orthodontics [17,18].

"Gummee Teething" and "The Teething Company," both of which are teething toy pages, demonstrated noteworthy engagement rates of 2.68% and 2.6%, respectively. This observation suggests that certain content strategies may resonate more effectively with users [19]. By offering tangible solutions to teething discomfort, these pages may particularly appeal to parents seeking remedies. Furthermore, their likely visually rich content could enhance user engagement.

However, it is worth noting that the absence of statistically significant differences in engagement rates across various groups/pages indicates an inconsistency in the effectiveness of content strategies on user engagement [15,18,20]. This underscores the complexity of user engagement behavior and emphasizes the need for further research to understand the elements that most effectively drive engagement.

The significant correlation between the type of posts and specific Facebook pages/groups ‎emphasizes how the nature of content influences user interaction [6]. These results advocate for ‎an increased proactive presence of healthcare professionals on social media, employing ‎strategies that include visual aids to improve user engagement [6,19,21,22] and combat the ‎proliferation of commercial and potentially misleading content.

The observed interactive engagement also signals a need for communal spaces where ‎caregivers can validate their experiences and gain access to accurate information, presenting a ‎prime opportunity for healthcare professionals to establish and manage moderated spaces that ‎offer sound guidance and support for effective teething management [6].

It is critical to acknowledge the limitations of this study. First, the standard engagement metrics such as likes, shares, and comments offer an incomplete picture of user engagement. They fail to capture passive consumption of information or how varied literacy levels impact interaction [1]. Additionally, different types of engagement might not be directly comparable, and a post with many comments could indicate either strong support or significant disagreement. This limitation, inherent to the use of engagement metrics, could affect the interpretation of user interactions or sentiment.

Second, cultural beliefs about teething may influence engagement with online content. A significant proportion of users (73%) may read posts without actively engaging, a phenomenon not detected through the current methodological lens [1].

Third, the study's inclusion criteria excluded pages and groups with restricted access, leading to potential selection bias. Such groups could contain useful and relevant discussions about teething, particularly in closed or private communities where users may feel more comfortable sharing personal experiences. This limitation could affect the generalizability of the findings and has been acknowledged as an area for potential future research.

Fourth, the sample size of the included groups and pages was relatively small, which may not adequately represent the full spectrum of available content on teething. The reasons for this low inclusion rate include strict adherence to the inclusion criteria and the quality of the available content. This small sample size could limit the generalizability of the findings.

Finally, the use of a single, newly created Facebook account for data collection may also introduce bias. Social media platforms such as Facebook personalize search results and content recommendations based on user search and behavior. As the account used was new, it could have limited the type and amount of content displayed. This limitation, which could affect the range and diversity of data collected, is acknowledged and emphasizes the need for caution in interpreting and generalizing the results.

## Conclusions

This study underscores the active community discussions about teething on Facebook, wherein ‎interactive content represents 58% of all posts. However, despite a considerable volume of ‎interactions, the engagement rate remains low at just 1.09%. This low rate suggests that, while ‎there is a notable quantity of interactions, they represent a small fraction of the total viewership, ‎indicating that most users tend to passively consume content without actively engaging.‎ Additionally, there is a critical shortage of authoritative, evidence-based information on ‎teething signs, symptoms, and relief practices, with misinformation about teething relief ‎practices being particularly evident.‎

These observations highlight the pressing need for healthcare professionals and children's ‎health organizations to provide accurate, scientifically supported information about teething. ‎Such efforts are essential to counter misinformation and cultivate a more informed and ‎proactive online community.‎

## References

[1] Rivera YM, Moran MB, Thrul J, Joshu C, Smith KC (2022). Contextualizing engagement with health information on Facebook: using the social media content and context elicitation method. J Med Internet Res.

[2] Kallem S, Gruver RS, Virudachalam S, Fiks AG (2018). Mothers' Facebook posts about infant health: findings from the Grow2Gether study. BMC Pediatr.

[3] Hassona Y, Abu Ghosh M, Abu Ghlassi T, Scully C (2016). Public engagement with oral cancer on Facebook. Oral Oncol.

[4] Stellefson M, Paige S, Apperson A, Spratt S (2019). Social media content analysis of public diabetes Facebook groups. J Diabetes Sci Technol.

[5] Pócs D, Adamovits O, Watti J, Kovács R, Kelemen O (2021). Facebook users’ interactions, organic reach, and engagement in a smoking cessation intervention: content analysis. J Med Internet Res.

[6] Olinski M, Szamrowski P (2021). Facebook as an engagement tool: how are public benefit organizations building relationships with their public?. PLoS One.

[7] Alkhozaim DA, Al-Haj Ali SN, Farah RI (2022). Levels and correlates of knowledge of teething among Saudi Arabian families. PeerJ.

[8] Jorge OS, Remiro MD, Lotto M, Zakir Hussain I, Moreira MA, Morita PP, Cruvinel T (2024). Unveiling deception: characterizing false amber necklace messages on Facebook. Int J Paediatr Dent.

[9] Wake M, Hesketh K, Allen M (1999). Parent beliefs about infant teething: a survey of Australian parents. J Paediatr Child Health.

[10] American Academy of Pediatric Dentistry (2023). Perinatal and infant oral health care. The Reference Manual of Pediatric Dentistry.

[11] Pereira TS, da Silva CA, Quirino EC (2023). Parental beliefs in and attitudes toward teething signs and symptoms: a systematic review. Int J Paediatr Dent.

[12] Cota AL, Silva EA, Freitas NB, Bisneto JS, Buriti GM, Valente JQ, Nemezio MA (2022). Use of the amber teething necklace by the child population: risks versus benefits. Rev Paul Pediatr.

[13] Ashley MP (2001). It's only teething...a report of the myths and modern approaches to teething. Br Dent J.

[14] Owais AI, Zawaideh F, Al-Batayneh OB (2010). Challenging parents' myths regarding their children's teething. Int J Dent Hyg.

[15] Abu-Ghazaleh S, Hassona Y, Hattar S (2018). Dental trauma in social media-analysis of Facebook content and public engagement. Dent Traumatol.

[16] Al-Haj Ali SN, Alsineedi F, Alsamari N, Alduhayan G, BaniHani A, Farah RI (2021). Risk factors of early childhood caries among preschool children in Eastern Saudi Arabia. Sci Prog.

[17] Fox K, Singh P (2022). What are dental professionals posting on Facebook? A cross-sectional content analysis. J Orthod.

[18] Alkadhimi A, Al-Moghrabi D, Alshehri RD, Watton M, Fleming PS (2022). The reach, influence and tenor of professional orthodontic societies on social media: a cross-sectional content analysis. Int Orthod.

[19] A Rahim AI, Ibrahim MI, A Salim FN, Ariffin MA (2019). Health information engagement factors in Malaysia: a content analysis of Facebook use by the Ministry of Health in 2016 and 2017. Int J Environ Res Public Health.

[20] Kim WB, Marinas JE, Vender RB (2015). Public engagement with dermatology contents on Facebook. J Cutan Med Surg.

[21] Strekalova YA, Krieger JL (2017). A picture really is worth a thousand words: public engagement with the National Cancer Institute on social media. J Cancer Educ.

[22] Gabarron E, Larbi D, Dorronzoro E, Hasvold PE, Wynn R, Årsand E (2020). Factors engaging users of diabetes social media channels on Facebook, Twitter, and Instagram: observational study. J Med Internet Res.

